# Isotope sample preparation of diatoms for paleoenvironmental research

**DOI:** 10.1371/journal.pone.0281511

**Published:** 2023-02-17

**Authors:** George E. A. Swann, Andrea M. Snelling

**Affiliations:** School of Geography, University of Nottingham, Nottingham, United Kingdom; University of Palermo: Universita degli Studi di Palermo, ITALY

## Abstract

Isotopes in diatoms are increasingly used in palaeoenvironmental studies in both lacustrine and marine settings, enabling the reconstruction of a range of variables including temperature, precipitation, salinity, glacial discharge, carbon dynamics and biogeochemical cycling. This protocol details an optimised methodology for extracting diatoms for isotope analysis from sediment samples, using a range of chemical and density separation techniques that minimise sample loss and avoids the need for expensive equipment. Whilst designed for the extraction of diatoms for oxygen, silicon and carbon isotope analysis, additional stages are outlined for the analysis of other isotopes that are of increasing interest to the palaeo community (e.g., boron and zinc). The protocol also includes procedures for assessing sample purity, to ensure that analysed samples produce robust palaeoenvironmental reconstruction. Overall, the method aims to improve the quality of palaeoenvironmental research derived from isotopes in diatoms by maximising sample purity and the efficiency of the extraction process.

## Introduction

Isotopes in diatoms (e.g., δ^13^C, δ^15^N, δ^18^O, δ^30^Si) provide a key source of palaeoenvironmental information in marine and lacustrine environments where carbonates not readily preserved in the sedimentary environment [1, 2]. Whilst the emergence of isotopes in diatoms as a palaeoenvironmental proxy has occurred alongside the development of mass-spectrometry techniques for their analysis [3–10], projects are often hindered by difficulties in extracting sufficient diatoms for analysis without the presence of non-diatom contaminants. Here we describe a protocol, suitable for Masters and PhD students, that has been used at the University of Nottingham for over a decade to obtain pure diatom samples from a sediment matrix, before samples are analysed at the National Environmental Isotope Facility (British Geological Survey) for δ^13^C, δ^18^O and δ^30^Si [6, 9]. Extensions/deviations to the core methodology are also outlined for samples that will be analysed for other/novel isotope systems that are of increasing interest to the palaeo community such as δ^11^B [11] and δ^66^Zn [12]. This protocol is not fully compatible with accepted diatom protocols for δ^15^N. Instead, samples for diatom δ^15^N should be prepared following [5, 13]. Caution should also be exerted when applying this, or indeed any, protocol to living/cultured diatom frustules due to the potential for post‐mortem oxygen isotope exchange [14].

Typically, in our experience, only 50–70% of sediment samples can be sufficiently cleaned to remove non-diatom contaminants and generate enough material for isotope analysis. This success rate varies between sites and different aged samples and is predominantly determined by the amount of raw material available, the concentration of diatoms and the presence/abundance of other types of biogenic silica (e.g., siliceous sponges and radiolaria) which can be problematic to separate. The protocol presented here has been tested on a wide variety of different aged lacustrine and marine sediments (0–3.4 Ma) and optimised to minimise the risk of sample loss. It also outlines stages for assessing and quantifying sample purity, as well as for checking that the extracted diatoms are not contaminated by diagenesis, dissolution or other processes that might have altered the isotopic signature.

## Materials and methods

The protocol described in this peer-reviewed article is published on protocols.io, https://dx.doi.org/10.17504/protocols.io.36wgq4knovk5/v2 and is included for printing as S1 File with this article.

## Expected results

**Fig 1:**
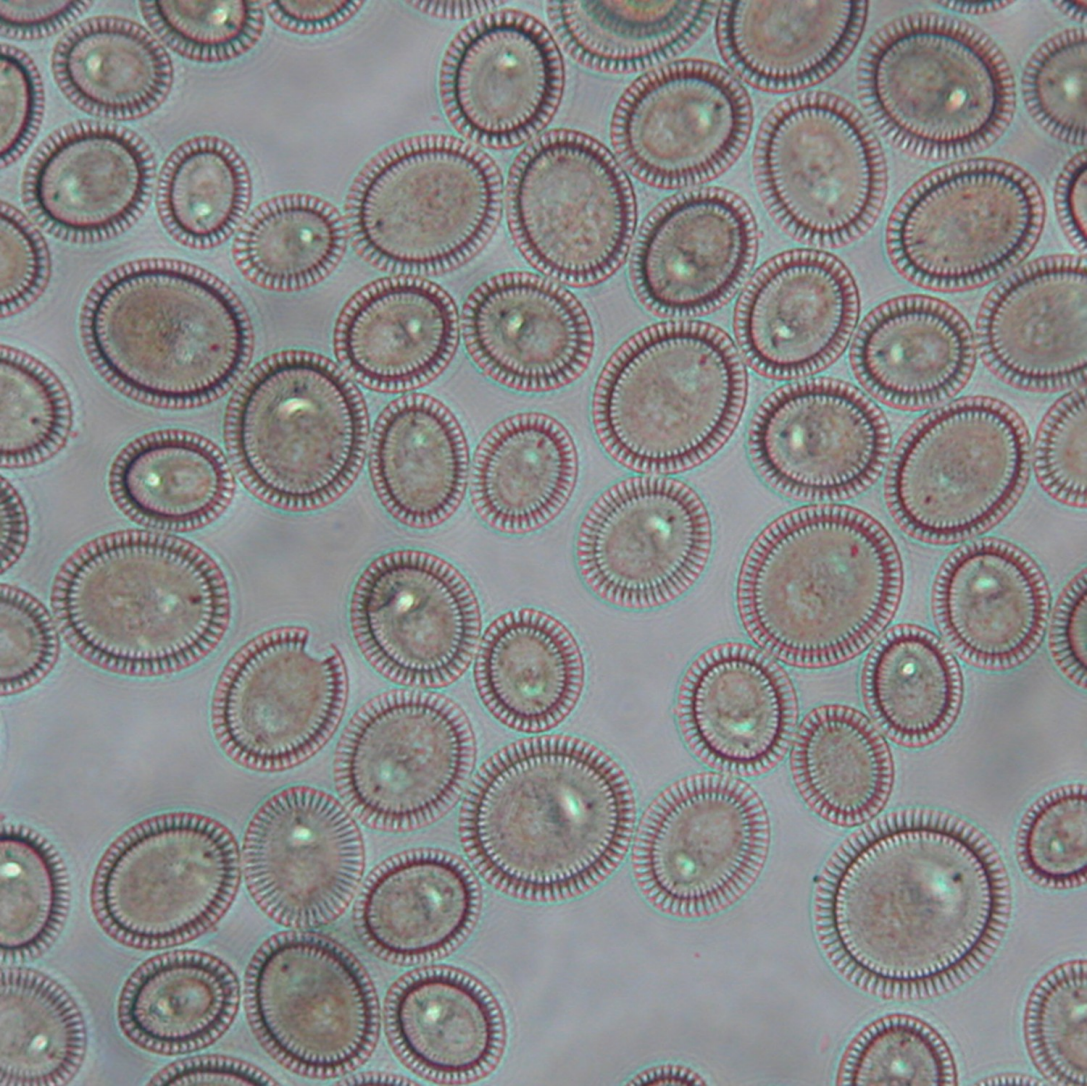
Example of a purified diatom sample (under light microscope) following the use of this protocol. Sample from Lake El’gygytgyn, Russia [15].

**Fig 2:**
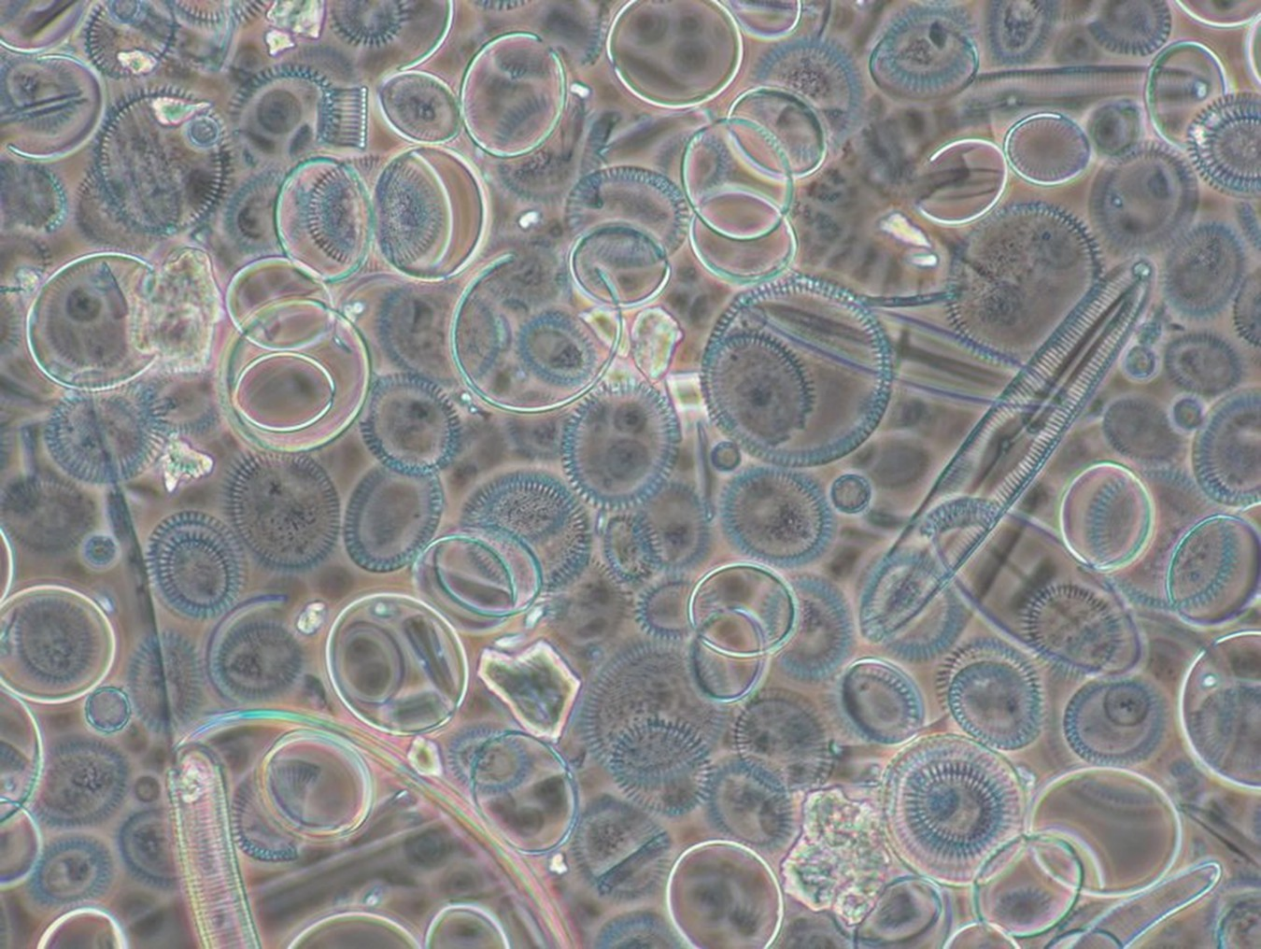
Example of a diatom sample (under light microscope) contaminated with aluminosilicates. Sample from Lake El’gygytgyn, Russia [15].

**Fig 3:**
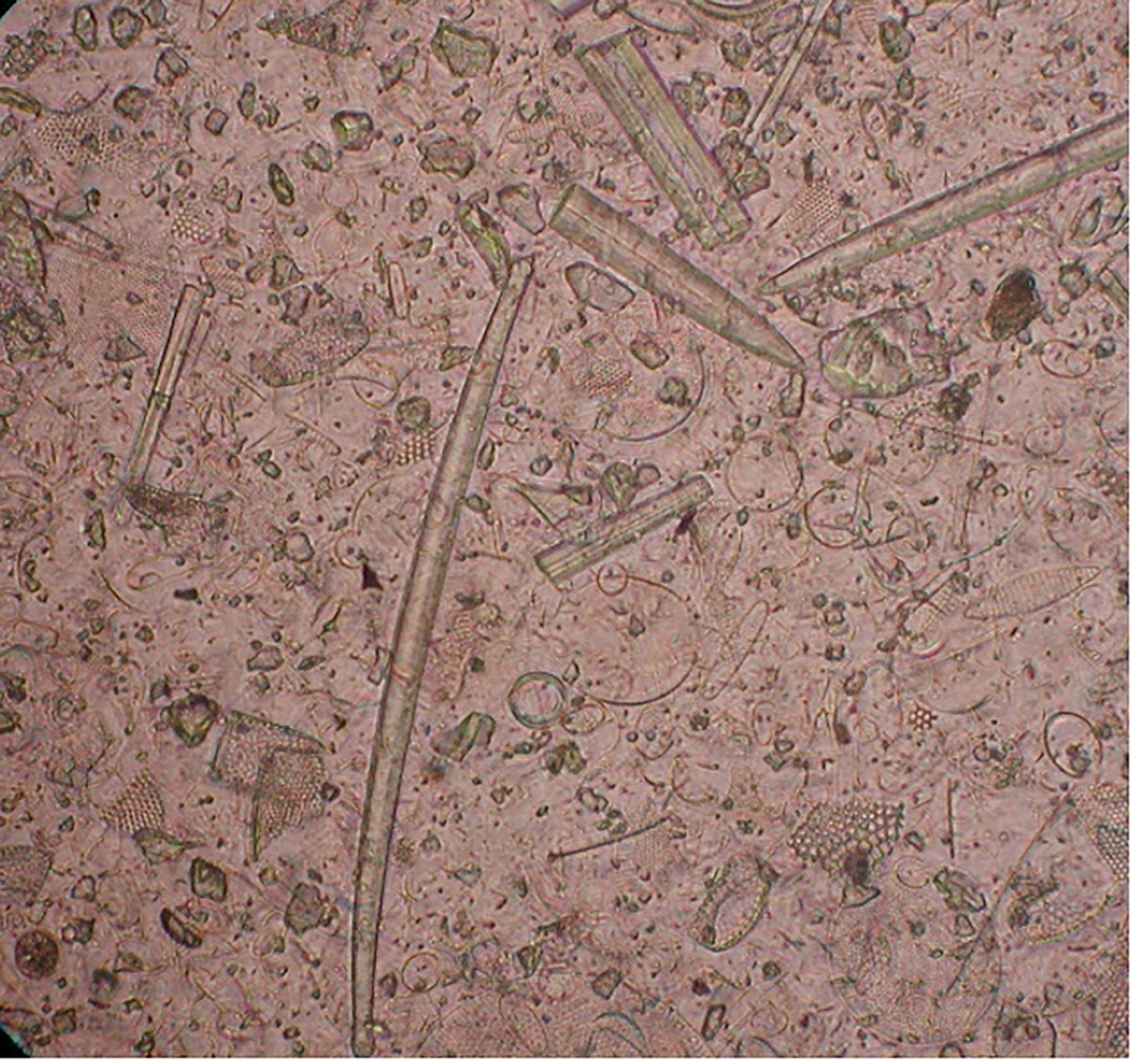
Example of a diatom sample (under light microscope) contaminated with aluminosilicates and sponge spicules. Sample from the Southern Ocean [17].

Using this protocol, we have been able to obtain pure diatom samples for isotope analysis with minimal material loss (Fig 1). The procedure has been successfully used on raw sediment samples as low as 0.5 g and 5–20% opal, with 6.5 mg of pure diatom needed for δ^18^O and δ^30^Si analysis [15]. In contrast, larger raw sediment samples have enabled the recovery of >20 mg pure diatoms and so permitted the analysis of δ^13^C [16], which typically requires larger amounts of material. As outlined in the protocol it is also possible, using different sized sieves and/or targeting laminated sediments, to obtain seasonal and/or intra-annual reconstructions [17] or other forms of biogenic silica (e.g., siliceous sponges and radiolaria). Whilst some sediment samples will not be "cleanable" due to the low diatom content, small sample size or inability to remove non-diatom contaminants (Figs 2 and 3), the use of contamination assessment techniques in the protocol allows sample purity to be quantified and ensures that affected samples are not inadvertently used in palaeoenvironmental reconstructions.

## Supporting information

S1 File Isotope sample preparation of diatoms for paleoenvironmental research, also available on protocols.io. **https://dx.doi.org/10.17504/protocols.io.36wgq4knovk5/v2**. The individual pictured in the S1 File has provided written informed consent (as outlined in PLOS consent form) to publish their image alongside the manuscript.
(PDF)

## References

[1] Leng MJ, Barker PA. A review of the oxygen isotope composition of lacustrine diatom silica for palaeoclimate reconstruction. Earth-Sci Rev. 2006; 75: 5–27.

[2] Swann GEA, Leng MJ. A review of diatom δ^18^O in palaeoceanography. Quat Sci Rev. 2009; 28: 384–398.

[3] de la Rocha CL, Brzezinski MA, DeNiro MJ. Purification, recovery, and laser-driven fluorination of silicon from dissolved and particulate silica for the measurement of natural stable isotope abundances. Anal Chem. 1996; 68: 3746–3750. doi: 10.1021/ac960326j 21619245

[4] de la Rocha CL. Measurement of silicon stable isotope natural abundances via multicollector inductively coupled plasma mass spectrometry (MC-ICP-MS). Geochem Geophy Geosy. 2002; 3: doi: 10.1029/2002GC000310

[5] Robinson RS, Brunelle BG, Sigman DM. Revisiting nutrient utilization in the glacial Antarctic: Evidence from a new method for diatom-bound N isotopic analysis. Paleoceanography. 2004; 19: PA3001, doi: 10.1029/2003PA000996

[6] Leng MJ, Sloane HJ. Combined oxygen and silicon isotope analysis of biogenic silica. J Quaternary Sci. 2008; 23: 313–319.

[7] Chapligin B, Meyer H, Friedrichsen H, Marent A, Sohns E, Hubberten H-W. A high-performance, safer and semi-automated approach for the δ^18^O analysis of diatom silica and new methods for removing exchangeable oxygen. Rapid Commun Mass Spectrom. 2010; 24: 2655–2664. 20740543 10.1002/rcm.4689

[8] Dodd JP, Sharp ZD. A laser fluorination method for oxygen isotope analysis of biogenic silica and a new oxygen isotope calibration of modern diatoms in freshwater environments. Geochim Cosmochim Acta. 2010; 74: 1381–1390.

[9] Hurrell ER, Barker PA, Leng MJ, Vane CH, Wynn P, Kendrick CP. et al. Developing a methodology for carbon isotope analysis of lacustrine diatoms. Rapid Commun Mass Spectrom. 2011; 25: 1567–1574. doi: 10.1002/rcm.5020 21594931

[10] Menicucci AJ, Matthews JA, Spero HJ. Oxygen isotope analyses of biogenic opal and quartz using a novel microfluorination technique. Rapid Commun Mass Spectrom. 2013; 27: 1873–1881. doi: 10.1002/rcm.6642 23857933

[11] Donald HK, Foster GL, Fröhberg N, Swann GEA, Poulton AJ, Moore CM, et al. The pH dependency of the boron isotopic composition of diatom opal (*Thalassiosira weissflogii*). Biogeosciences. 2020; 17: 2825–2837.

[12] Andersen MB, Vance D, Archer C, Anderson RF, Ellwood MJ, Allen CS. The Zn abundance and isotopic composition of diatom frustules, a proxy for Zn availability in ocean surface seawater. Earth Planet Sc Lett. 2011; 301: 137–145.

[13] Studer AS, Sigman DM, Martínez-García A, Thöle LM, Michel E, Jaccard SL, et al. Increased nutrient supply to the Southern Ocean during the Holocene and its implications for the pre-industrial atmospheric CO_2_ rise. Nat Geosci. 2018; 11: 756–760.

[14] Tyler J.J., Sloane H.J., Rickaby R.E.M., Cox E.J., Leng M.J. Post‐mortem oxygen isotope exchange within cultured diatom silica. Rapid Communications in Mass Spectrometry. 2017; 31: 1749–1760. doi: 10.1002/rcm.7954 28792631 PMC5639378

[15] Swann GEA, Leng MJ, Juschus O, Melles M, Brigham-Grette J, Sloane HJ. A combined oxygen and silicon diatom isotope record of Late Quaternary change in Lake El’gygytgyn, North East Siberia. Quat Sci Rev. 2010; 29: 774–786.

[16] Swann GEA, Kendrick CP, Dickson AJ, Worne S. Late Pliocene marine *p*CO_2_ reconstructions from the subarctic Pacific Ocean. Paleoceanography and Paleoclimatology. 2018; 33: 457–469.

[17] Swann GEA, Pike J, Snelling AM, Leng MJ, Williams, MC. Seasonally resolved diatom δ^18^O records from the West Antarctic Peninsula over the last deglaciation. Earth Planet Sci Lett. 2013; 364: 12–23.

