# Supplementary material for: Isotope sample preparation of diatoms for paleoenvironmental research
Source: PLoS One. 2023 Feb 17;18(2):e0281511. doi: 10.1371/journal.pone.0281511 (PMC9937473; doi:10.1371/journal.pone.0281511)
Supplement: S1 File — https://dx.doi.org/10.17504/protocols.io.36wgq4knovk5/v2. The individual pictured in the S1 File has provided written informed consent (as outlined in PLOS consent form) to publish their image alongside the manuscript. (PDF) [file pone.0281511.s001.pdf]

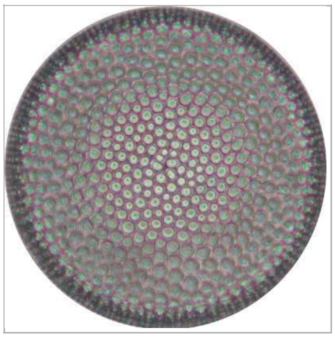

Isotope sample preparation of diatoms for paleoenvironmental research V.2

George Swann[1], Andreasnelling[1]

[1]University of Nottingham

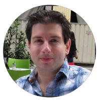

George Swann
University of Nottingham

VERSION 2
JAN 03, 2023

OPEN ACCESS

DOI:
dx.doi.org/10.17504/protocols.io.36wgq4knovk5/v2

Protocol Citation: George Swann, Andreasnelling 2023. Isotope sample preparation of diatoms for paleoenvironmental research. **protocols.io** https://dx.doi.org/10.17504/protocols.io.36wgq4knovk5/v2
Version created by George Swann

License: This is an open access protocol distributed under the terms of the Creative Commons Attribution License, which permits unrestricted use, distribution, and reproduction in any medium, provided the original author and source are credited

Protocol status: Working
We use this protocol and it's working

Created: Jan 03, 2023

Last Modified: Jan 03, 2023

PROTOCOL integer ID: 74687

Keywords: biogenic silica, opal, geochemistry, paleoclimate, paleoceanography, paleolimnology

ABSTRACT

Isotopes in diatoms are increasingly used in palaeoenvironmental studies in both lacustrine and marine settings, enabling the reconstruction of a range of variables including temperature, precipitation, salinity, glacial discharge, carbon dynamics and biogeochemical cycling. This protocol details an optimised methodology for extracting diatoms for isotope analysis from sediment samples, using a range of chemical and density separation techniques that minimise sample loss and avoids the need for expensive equipment. Whilst designed for the extraction of diatoms for oxygen, silicon and carbon isotope analysis, additional stages are outlined for the analysis of other isotopes that are of increasing interest to the palaeo community (e.g., boron and zinc). The protocol also includes procedures for assessing sample purity, to ensure that analysed samples produce robust palaeoenvironmental reconstruction. Overall, the method aims to improve the quality of palaeoenvironmental research derived from isotopes in diatoms by maximising sample purity and the efficiency of the extraction process.

GUIDELINES

This protocol has been designed and tested with a range of different aged marine and lacustrine sediments that have either been "wet" or freeze dried before use. The technique is suitable for all lab users, including masters and PhD students. Whilst the order of Steps 1-7 can be changed, the order listed here is the most efficient in terms of time and maximising the amount of diatom frustules recovered from any sample.

The protocol has been routinely used for diatom $\delta^{13}C$, $\delta^{18}O$, $\delta^{30}Si$ analysis and can be extended (as outlined in Stage 9.5) for other isotopes including $\delta^{11}B$ and $\delta^{66}Zn$. However, this protocol is not fully compatible with accepted diatom isotope protocols for $\delta^{15}N$. Instead, samples for diatom $\delta^{15}N$ should be prepared following Robinson et al. (2004) and Studer et al., (2018). Caution should be exerted when applying this, or indeed any, protocol to living/cultured diatom frustules due to the potential for post-mortem oxygen isotope exchange (Tyler et al., 2017).

Although not designed for this purpose, the protocol can also be used to extract other forms of biogenic silica, (e.g., siliceous sponges and radiolaria).

MATERIALS TEXT

Equipment for diatom extraction

- Centrifuge
- Deionised water
- Fume cupboard
- Pasteur pipette (3 ml)
- Pipette (1 ml) and pipette tips
- Plastic Centrifuge tubes (10-15 ml)
- Plastic containers for storing sodium polytungstate solution prior to use
- Test tube racks

Chemicals for diatom extraction

- H_2O_2
- HCl
- Sodium polytungstate (SPT)

Equipment for SPT recycling (see Step 9 or protocol)

- Cellulose nitrate membrane filters (5 µm, 1 µm and 0.45 µm)
- Four empty containers (suggested capacity is 2.5 litres) for storing SPT waste labelled #1, #2, #3 and #4.
- 2x HDPE plastic flask with side arm (1 litre) or item similar to this.
- Pump (hand or vacuum)
- Upper flask chamber with locking ring and filter support plate

Optional equipment for assessing sample purity (see Step 8 of protocol)

- Fourier Transform Infrared (FTIR) Spectrometer
- Light microscope (x400 and x1000 magnification)
- Inverted Microscope (≥ x400 magnitficaton)
- Scanning Electon Microscope
- XRF

SAFETY WARNINGS

Ensure appropriate personal protective equipment is worn at all times when handling chemicals, reagents and samples.

Disaggregation of samples

3h 30m

1

This step breaks up aggregated sediment using non-alkaline chemicals so that diatoms can be successfully extracted. Full removal of external organic matter occur later in Step 6 of the protocol.

1.1 Place up to 1 cm^3 of freeze dried sediment sample in 10-12 ml centrifuge tube labelled with "A" suffix.

Note

Weight should be adjusted accordingly if using material that has not been freeze dried.

1.2 Add 0.5 mL of [M] 30 % volume H_2O_2 at Room temperature in a fume cupboard and allow to react for 00:30:00 30m

Safety information

Monitor in case samples react vigorously. Add deionised water (or equivalent) if samples are at risk of over-spilling the centrifuge tube.

1.3 Add a further 0.5 mL of [M] 30 % volume H_2O_2 at Room temperature in a fume cupboard and allow to react for 03:00:00 3h

Safety information

Monitor in case samples react vigorously. Add deionised water (or equivalent) if samples are at risk of over-spilling the centrifuge tube.

Note

Adding this second aliquot of H2O2 ensures that samples are disaggregated. It is not added earlier to avoid samples over-reacting and potentially spilling out of the centrifuge tube.

1.4 Add deionised water (or equivalent) to top of centrifuge tube and shake to combine, ensuring that all of the

sample is in suspension. Leave Overnight in a fume cupboard.

Safety information

Ensure sample lids are loosened overnight to prevent the build up of gases.

1.5 Tighten lids and 1500 rpm, 00:05:00 5m

1.6 Syphon off solution/suspended material.

Note

Fine material in suspension will not contain diatoms and can be safely discarded.

1.7 Second wash: fill centrifuge tube to top with deionised water (or equivalent) and shake to combine, ensuring that all of the sample is in suspension. Repeat steps 1.5 and 1.6.

1.8 Third wash: fill centrifuge tube to top with deionised water (or equivalent) and shake to combine, , ensuring that all of the sample is in suspension. Repeat steps 1.5 and 1.6.

Note

After third wash ensure as much water is syphoned off as possible.

SPT: 1st separation

20m

2 **This step aims to separate diatoms from clay/aluminosilicates. A range of heavy density liquids are available, however this protocol uses sodium polytungstate (SPT) which:**

- **is a non-toxic high density agent;**
- **creates a non-alkaline solution when dissolved in water;**
- **can easily be recovered and recycled for future use.**

Safety information

Ensure appropriate dust mask is worn when handling SPT in dry form to avoid inhalation.

2.1 Make up heavy liquid SPT solution to achieve a specific gravity of 2.25-2.30. Ensure SPT powder is fully dissolved before use - solution should be transparent.

Note

Specific gravity of diatoms is typically c. 2.1.

2.2 Add 4 mL of SPT solution to each centrifuge tube and shake to combine, ensuring that all of sample is in suspension. 2500 rpm, 00:20:00 20m

Note

After centrifuging diatoms should be concentrated in the supernate and clays in the precipitate. However, it is normal at this point for some clays to be trapped in the supernate and for some diatoms to remind in the precipitate.

2.3 Label a second centrifuge tube with the suffix ‘B’

2.4 Add a small amount of deionised water (or equivalent) to the top of the "A" centrifuge tube and use a 3 ml Pasteur pipette to extract the supernate and as much SPT solution as possible into the ‘B’ centrifuge tube without disturbing the precipitate.

Note

The addition of a small amount of water helps prevent diatoms from sticking to the side of the centrifuge tube.

2.5 Add 4 mL of SPT solution to the "A" centrifuge tube and shake to combine, ensuring that all of the sample is in suspension.

Note

This second SPT wash of the "A" centrifuge tube is to recover diatoms that may have become trapped in the precipitate. In our experience, this second SPT wash can be important for maximising diatom extraction in some samples, whilst in other samples it does not lead to the recovery of any additional diatom frustules. In some samples we have noticed that a significant number of diatoms are not present in the supernate until after this second SPT wash.

2.6 2500 rpm, 00:20:00 20m

2.7 Repeat step 2.4

2.8 Fill "B" centrifuge tube to the top with deionised water (or equivalent) and shake to combine, ensuring that all of the sample is in suspension. 1500 rpm, 00:05:00 5m

Note

After centrifuging, diatoms will be in the precipitate. Everything in suspension is SPT and/or non-diatom contaminants.

2.9 Syphon liquid suspension in "B" centrifuge tube into SPT waste container #1 for recycling (see Section 9).

SPT: 2nd separation

25m

3 **The SPT separation is repeated for a second time with minor differences to the first SPT separation.**

3.1 Add 4 mL of SPT solution and 0.15 mL deionised water (or equivalent) to "B" centrifuge tube and shake to combine. Ensure all of sample is in suspension. 2500 rpm, 00:20:00 20m

Note

Adding this additional water to the sample lowers the specific gravity of the solution, increasing the separation of diatoms from clays.

3.2 Label a second centrifuge tube with the suffix 'C'

3.3 Add a small amount of deionised water (or equivalent) to the top of the "B" centrifuge tube and use a 3 ml Pasteur pipette to extract the supernate and as much SPT solution as possible into the 'C' centrifuge tube without disturbing the precipitate.

3.4 Fill "C" centrifuge tube to the top with deionised water (or equivalent) and shake to combine, ensuring that all of the sample is in suspension. 1500 rpm, 00:05:00 5m

Note

After centrifuging, diatoms will be in the precipitate. Everything in suspension is SPT and/or non-diatom contaminants.

3.5 Syphon liquid suspension in "C" centrifuge tube into SPT waste container #1 for recycling (see Section 9).

SPT: 3rd separation

25m

4 **The SPT separation is repeated for a third time with minor differences to previous SPT separations.**

4.1 Add 4 mL of SPT solution and 0.3 mL deionised water (or equivalent) to "C" centrifuge tube and shake to combine. Ensure all of sample is in suspension. 2500 rpm, 00:20:00 20m

Note

Adding this additional water to the sample lowers the specific gravity of the solution, increasing the separation of diatoms from clays.

4.2 Label a second centrifuge tube with the suffix 'D'

4.3 Add a small amount of deionised water (or equivalent) to the top of the "C" centrifuge tube and use a 3 ml Pasteur pipette to extract the supernate and as much SPT solution as possible into the 'D' centrifuge tube without disturbing the precipitate.

4.4 Fill "D" centrifuge tube to the top with deionised water (or equivalent) and shake to combine, ensuring that all of the sample is in suspension. 1500 rpm, 00:05:00 5m

Note

After centrifuging, diatoms will be in the precipitate. Everything in suspension is SPT and/or non-diatom contaminants.

4.5 Syphon liquid suspension in "D" centrifuge tube into SPT waste container #1 for recycling (see Section 9).

4.6 First wash: fill "D" centrifuge tube to top with deionised water (or equivalent). Shake to combine, ensuring all material is in suspension. 1500 rpm, 00:05:00 . 5m

4.7 Syphon off solution/suspended material.

4.8 Repeat steps 4.6 and 4.7 for a second wash.

SPT residue clean-up

5 **Material left in the "A", "B" and "C" centrifuge tubes will contain non-diatom contaminants. Residue SPT liquid in these samples should be recovered for recycling.**

5.1 Add 10 mL of deionised water (or equivalent) to "A", "B", and "C" centrifuge tubes and shake to combine, ensuring that all of the sample is in suspension. 1500 rpm, 00:05:00 5m

5.2 Syphon liquid (including material in suspension) into the SPT waste container #1 for recycling (see Section 9).

5.3 Repeat steps 5.2 and 5.3 twice

Note

Remaining material will be comprised of non diatom-contaminants. If required, this material can be retained for future analysis and/or analysed to understand the isotope/geochemical composition of non-diatom contaminants.

Organics removal

1w 0d 0h 10m

6 **At this point the remaining sample material in the "D" centrifuge tube should be mainly comprised of diatoms that are potentially covered in external organic matter. This step removes this organic matter using H_2O_2. Whilst others have used stronger chemicals such as nitric acid, perchloric acid (see Tyler et al., 2007) or even hydrogen fluoride, we elect to use H_2O_2 as it is typically sufficient for removing all organic matter and minimises the risks of attacking the diatom frustules.**

6.1 Add 10 mL H_2O_2 to “D" centrifuge tube and shake to combine all material. Loosen lids and place in heat 1w

block/water bath at 75 °C in a fume cupboard. Leave for 168:00:00 (1 week), topping up centrifuge tubes with H_2O_2 where necessary.

Safety information

Ensure sample lids are still loosened once the centrifuge tubes have warmed up in the water bath to prevented the build up of gases.

6.2 Allow samples to cool to Room temperature . Tighten lids and 1500 rpm, 00:05:00 . 5m

6.3 Syphon off solution and fill centrifuge tube to the top with deionised water (or equivalent). Shake centrifuge tube to combine, ensuring that all of the sample is in suspension. 1500 rpm, 00:05:00 . 5m

6.4 Repeat Step 6.3 twice.

6.5 Syphon off solution.

Carbonate removal

5m

7 **Samples at this point will typically contain no/minimal amounts of carbonate. However, this step remains important to ensure that all trace levels of carbonate are fully removed.**

7.1 Add [M] 5 % volume HCl to top of centrifuge tube. Shake to combine, ensuring all material is in suspension. Leave Overnight at Room temperature in a fume cupboard.

Safety information

Ensure sample lids are loosened to prevent the build up of gases.

7.2 Syphon off solution.

7.3 Fill centrifuge tube to top with deionised water (or equivalent). Shake to combine, ensuring all material is in suspension. 1500 rpm, 00:05:00 . 5m

7.4 Repeat step 7.2 and 7.3 twice for second and third washes.

7.5 Syphon off solution.

Purity assessment

8 **At this point in the protocol, an assessment of sample purity is recommended to identify what additional work is required. This also allows the diatom frustules to be checked for evidence of dissolution, diagenesis or other processes that might have caused isotope fractionation.**

8.1 **Initial assessment:** Check sample purity under a light microscope (using a smear slide) or inverted microscope [in a plastic petri dish] at ≥ x400 magnification. Contamination can be assessed by eye and/or through quantitative counts using a grid graticule (see Morley et al., 2004).

- If sample is "contaminated", go to Step 8.2.
- If sample appears clean, go to Step 8.3 for further assessment of sample purity.

8.2 If sample is contaminated, conduct further clean-up steps based on the type of contaminant in the sample. 5m

Clay/aluminosilicate contamination: complete the sub-steps below in the order listed. Perform an "initial assessment of sample purity" (Step 8.1) between each sub-step to check whether the contaminants have been removed.

- Repeat 3rd SPT wash ( )
- Differential settling (see Morley et al. (2004))
- Sieve sample. In addition to removing contaminants, sieving samples can be useful to remove other forms of biogenic silica and/or separate diatom frustules/species which have different sizes. It is recommended that: 1) prior to sieving, careful visual analysis of the sample under a

microscope is carried out to identify what size sieve/sieve cloth is used; and 2) to avoid loss of material, all sieving is done over a 0.45 µm cellulose nitrate membrane filter. This filter can be rolled up and place into the top of a centrifuge tube, filled with deionised water (or equivalent) and 1500 rpm, 00:05:00 to recover the material off the filter.

Organic matter

- Repeat organic removal ( )

Other biogenic silica (e.g., radiolaria, sponges)

- Sieve sample (see above)

After further cleaning . If visual inspections show sample can not be purified, either:

- disregard for isotope analysis;
- consider separating diatoms/contaminants using a micro-manipulator (Snelling et al., 2013). As the use of a micro-manipulator is time consuming, it is recommended that this only be done on critical samples.

8.3 **Further assessments of sample purity:** There are a number of options for this and it is suggested that at least one type of assessment is carried out.

- Light microscope (x1000 magnification) using a grid graticule to quantify contamination (Morley et al., 2004).
- XRF - this was originally done by Brewer et al. (2008) who used the data to mass-balance the isotopic impact of non-diatom contamination in samples. We now suggest that XRF is simply used to check sample purity (rather than as the basis for mass-balancing) with contaminated samples either undergoing further purification or disregarded (see Step 8.2). At the University of Nottingham we analyse samples in XRF as a loose powder (not pressed pellet) to ensure material can be recovered afterwards for isotope analysis. As XRF provides a "whole sample" perspective of sample purity, we predominantly use it to check for clay/aluminosilicate contamination. Taking into account the presence of naturally occluded Al within diatom frustules, we require samples to have a Al/Si ratio of at least $\leq$0.03 and ideally $\leq$0.02 to be considered "clean".
- Scanning electron microscope (SEM) - this can identify micro-contaminants within a sample (e.g., Brewer et al. (2008)).
- SEM with Energy-Dispersive-X-ray Spectroscopy (EDS) - Chapligin et al. (2012).
- Inductively Coupled Plasma Optical Emission Spectroscopy (ICP-OES) - Chapligin et al. (2012).
- Fourier Transform Infrared (FTIR) - see Swann and Patwardhan (2011).

8.4 If samples are free of non-diatom contamination, samples can be analysed for $\delta^{13}C$, $\delta^{18}O$ $\delta^{30}Si$. For the analysis of other isotopes that have begun to be developed for diatoms, further steps may be required to remove trace organics. These additional steps are outlined in the citations below, but the need for these (or modification to them) may change as the analysis of these isotopes becomes more widespread.

- $\delta^{11}B$: Donald et al., 2020.
- $\delta^{66}Zn$: Andersen et al., 2011.

SPT filtering

9 **This protocol can generate significant amounts of SPT waste. However SPT can be easily recovered and purified for future use.**

9.1 Sieve SPT from waste container #1 through a 5 µm cellulose membrane and place into SPT waste container #2. Use a vacuum pump to expedite this process, although a hand pump can instead be used.

Note

Ensure that an overflow container is attached to prevent SPT entering pump.

9.2 Sieve SPT from waste container #2 through a 1 µm cellulose membrane and place into SPT waste container #3.

9.3 Sieve SPT from waste container #3 through a 0.45 µm cellulose membrane and place in into SPT waste container #4.

9.4 Place SPT solution into evaporating bowl and into drying cabinet (max temperature should be <105°C. Once dry, SPT can be ground and reused.

Note

Removing the dried SPT from the evaporating bowl is best achieved by hitting the SPT with a pestle. This can risk breaking the evaporating bowl if a thin bowl is used. Alternatively, the density of the SPT can monitored during the drying process until the solution reaches the specific gravity of 2.25-2.30 that is used in Step 2 of this protocol.
