# Supplementary material for: Isotope sample preparation of diatoms for paleoenvironmental research
Source: PLoS One. 2023 Feb 17;18(2):e0281511. doi: 10.1371/journal.pone.0281511 (PMC9937473; doi:10.1371/journal.pone.0281511)
Supplement: Attachment — Submitted filename: response to reviews.docx [file pone.0281511.s002.docx]

**“Isotope sample preparation of diatoms for paleoenvironmental research”**

**[PONE-D-22-32814]**

**Detailed responses to reviewers comments**

**Our responses below are in bold. All issues have been addressed with relevant text added as indicated. In addition, we have ensured that the manuscript is fully formatted in line with the PLOS ONE journal specific requirements.**

**Reviewer 1**

…I would argue that diatoms do not have isotopes, elements have isotopes. I would suggest that the authors change the title, and subsequently other parts of the manuscript where they use the phrase "diatom isotopes"… :

**We have changed the title of the manuscript and associated protocol (Supplementary Information) to “Isotope sample preparation of diatoms for paleoenvironmental research”. Text in other relevant sections of the manuscript have also been altered accordingly.**

Also, I wonder if you could not be more inclusive to suggest that this type of protocol could also be used for other silicifying organisms, such as sponges.

**We now state this in the “Expected results” section of the manuscript and in the protocol itself.**

**Reviewer 2**

Interestingly, the authors choose to heat the samples to 75C with H_2_O_2_ for removal of organic materials, but did not include use of nitric acid in their organic removal methodology. As they have mentioned, their protocol is designed specifically for stable isotope analysis (with the exception of δ15N); I am curious what the author’s reasons are for not choosing this acid to induce a more rapid oxidation reaction (faster than the recommended one week at 75C) with the organic materials. It is not essential, but among modern diatom ecologists, the use nitric acid is common... Nitric acid also removes carbonate contaminants from sediment samples.

**In the protocol (Supplementary Information) we have now made it clearer that our use of hydrogen peroxide (H_2_O_2_) over nitric acid (HNO_3_) is predominantly one of personal preference. We have made it clear that other chemicals (e.g., HNO_3_) exist to remove organi matter and cite the study of Tyler et al 2017 (**[**https://doi.org/10.1007/s10933-006-9030-9**](https://doi.org/10.1007/s10933-006-9030-9)**) who demonstrated the effectiveness of each option.**
